# Decreased brain-derived neurotrophic factor expression in chronic kidney disease: integrated clinical and experimental evidence

**DOI:** 10.3389/fmolb.2025.1627534

**Published:** 2025-07-28

**Authors:** Juan Chen, Lili Fu, Mengjin Li, Kun Xie, Xinming Li, Xu-jie Zhou, Li Yang, Liming Zhang, Cheng Xue, Zhiguo Mao

**Affiliations:** ^1^ Department of Nephrology, Shanghai Changzheng Hospital, Second Affiliated Hospital of Naval Medical University, Second Military Medical University, Shanghai, China; ^2^ Department of Nephrology, Northern Jiangsu People’s Hospital Affiliated to Yangzhou University, Yangzhou, Jiangsu, China; ^3^ Key Laboratory of Renal Disease, Peking University First Hospital, Renal Division, Ministry of Health of China, Institute of Nephrology, Peking University, Beijing, China; ^4^ Department of Cellular Biology and Anatomy, Medical College of Georgia at Augusta University, Augusta, GA, United States

**Keywords:** chronic kidney disease, brain-derived neurotrophic factor, meta-analysis, single-cell transcriptomics, Western blot

## Abstract

**Background:**

Chronic kidney disease (CKD) is a progressive disorder characterized by declining renal function and systemic metabolic disturbances. Brain-derived neurotrophic factor (BDNF), a key member of the neurotrophic family, plays critical roles in neuronal function and muscular metabolism. However, the evidence and regulatory mechanisms underlying decreased BDNF levels in CKD remain inconclusive.

**Methods:**

This study systematically evaluated circulating BDNF alterations in CKD patients through a meta-analysis of clinical studies involving 1,549 participants, complemented by experimental validation in unilateral ureteral obstruction (UUO) mice and single-cell transcriptomic database analysis to investigate tissue-specific BDNF protein expression and regulatory patterns.

**Results:**

Meta-analysis confirmed significantly reduced circulating BDNF in CKD patients (WMD = −0.62 ng/mL, 95% CI [-0.98, −0.25], *P* < 0.001; *I* ^*2*^ = 87%). In 14-day UUO mice, renal immunohistochemistry (IHC) showed significantly reduced BDNF expression (*P* < 0.001), which was further validated by Western blot analysis demonstrating a progressive decline in BDNF protein levels from day 14 to day 21 post-obstruction. Single-cell mRNA sequencing further confirmed that *Bdnf* levels were lower in renal proximal tubule (PT) cells, macrophages (Mφ), and podocytes in UUO mice compared to normal controls, Additionally, *Bdnf-as*—a long non-coding RNA known to epigenetically repress BDNF—was significantly upregulated in proximal tubules of CKD patients based on human transcriptomic data. This upregulation was validated in UUO mice by qPCR, showing a time-dependent increase in *Bdnf-as* expression at days 14 and 18 post-obstruction.

**Conclusion:**

This study integrated meta-analysis, murine model validation, and single-cell transcriptomic profiling to demonstrate a significant reduction of BDNF in CKD. Furthermore, renal BDNF expression decreased locally, predominantly originating from proximal tubule cells, macrophages, and podocytes, possibly epigenetically inhibited by the upregulation of lnc RNA *Bdnf-as*.

## Introduction

Chronic Kidney Disease (CKD) is a progressive condition that leads to kidney dysfunction and various systemic complications, including cardiovascular disease, metabolic disorders, neurodegenerative diseases, and anemia (Francis et al., 2024; Liu et al., 2023). Epidemiologically, CKD affects approximately 10%–15% of the global population, with a rising prevalence due to aging populations, diabetes, and hypertension (Bikbov et al., 2020). Cognitive impairment in CKD patients may be linked to the buildup of uremic toxins and oxidative stress (Van Sandwijk et al., 2016). As the disease advances, renal replacement therapies like dialysis or transplantation are often required (Van Sandwijk et al., 2016).

Brain-derived neurotrophic factor (BDNF) is an important neurotrophic factor widely expressed in the nervous system, where it regulates synaptic plasticity and neuronal survival (Wang et al., 2022). Its secretory function has also been observed in non-neural tissues, such as the kidneys (Kamel et al., 2025) and liver (Girard et al., 2021). BDNF is expressed in both glomeruli and renal tubules, while its receptors, TrkB and TrkC, are present in proximal and distal tubules as well as collecting duct epithelial cells. Through interaction with TrkB, BDNF plays a role in modulating local inflammation and maintaining metabolic homeostasis (Huber et al., 1996; Pei et al., 2024). Low serum BDNF levels have been linked to chronic CKD complications, including pruritus (Śnit et al., 2020) and cognitive impairment (Lee et al., 2018), though direct evidence of BDNF’s role in CKD is still lacking.

This study aimed to investigate changes in BDNF expression associated with CKD using a multidimensional approach, including (Francis et al., 2024) a meta-analysis of studies to assess serum BDNF reduction in CKD populations (Bikbov et al., 2020); unilateral ureteral obstruction (UUO) mouse models to investigate renal BDNF expression and pathology; and (Van Sandwijk et al., 2016) single-cell sequencing to detect BDNF expression and regulation.

## Methods

### Meta-analysis design

#### Literature search and study selection

We systematically searched PubMed for relevant studies up to 30 March 2025, with specific search strategies outlined in Supplementary Table S1. Following the Preferred Reporting Items for Systematic Reviews and Meta-Analyses (PRISMA) statement (Melkevik et al., 2010), we performed a meta-analysis following established methodological standards in Supplementary Table S2.

#### Inclusion and exclusion criteria

The inclusion criteria were as follows: 1) Study designs limited to epidemiological research, observational studies (retrospective or prospective), and controlled clinical trials; 2) Study population explicitly comprising CKD patients with serum/plasma BDNF measurements; 3) Reporting of BDNF-related statistical parameters including odds ratios (OR), relative risks (RR), mean ± Standard Deviation (SD), or 95% confidence intervals (95% CI). Exclusion criteria: 1) non-original research (reviews, guidelines, meta-analyses, editorials, case reports, commentaries, letters); 2) Animal models, and *in vitro* experiments; 3) Studies with incomplete data, non-convertible metrics, or fundamental design flaws compromising validity; 4) Unavailable full-text publications; 5) Lack of control groups. The selection of studies was done by CJ and XC.

#### Data extraction and quality assessment

Data were systematically extracted from the included studies by CJ and XC, including the following information: first author, publication year, country, study design, study setting, gender distribution (number of males and females), sample size each group, mean and standard deviation (SD) of serum or plasma BDNF levels, definition of CKD, and sample source.

Assessment of the risk of bias was performed by CJ and XC. This study utilized the cross-sectional study quality assessment tool developed by the Agency for Healthcare Research and Quality (AHRQ) to systematically evaluate the risk of bias in the included literature (Zeng et al., 2015). The tool comprises 11 criteria, with item 5 being reverse-scored (“yes = 0, no/unclear = 1”). All other items were positively scored (“yes = 1, no/unclear = 0”). Studies with a total score of ≥8 points were classified as high quality, those scoring 5–7 points as moderate quality, and those with <5 points as low quality.

### Animal experiments

#### Animals

SPF-grade C57BL/6J wild-type mice were purchased from Shanghai Jihui Laboratory Animal Breeding Base, 6–8 weeks male mice, weighing 23–25 g. They were directly sent to the SPF-grade mouse breeding center of the Naval Military Medical University to continue to be bred for 1–2 weeks at the following temperatures: 22°C ± 1°C, relative air humidity of about 50%–60%, guaranteed standard mouse food, free drinking water, and alternating cycles of light and darkness of 12 h per day.

#### UUO model protocol

An experimental animal protocol was approved at our institution, and mice were randomly divided into five groups: Sham surgery group, UUO surgery 7 days, 14 days, 18 days, and 21 days groups. Mice were weighed, anesthetized by intraperitoneal injection of 3% sodium pentobarbital (75 mg/kg), and immobilized in the supine position on a temperature-controlled pad at approximately 37°C. After disinfection with iodophor cotton balls, a 1.5-cm longitudinal incision was made along the white line of the abdomen, bluntly detached, and the ureter was double ligated (spaced at a distance of 2–3 mm) and cut in the middle, and the organs were reset and then the muscularis propria was sutured (continuous suture) to the skin (interrupted suture) in layers. In the sham group, the same surgery was performed without treating the kidneys and ureters. After surgery, 1 mL of saline was injected intraperitoneally into the mice to prevent dehydration.

#### Tissue collection

Animals were euthanized by cervical dislocation under anesthesia at the endpoint of observation at 7, 14, 18, 21 days post-infarction. Cardiac perfusion was performed until the kidneys turned white to obtain obstructed renal tissue, which was split longitudinally and fixed in 4% paraformaldehyde (for immunohistochemistry).

#### Immunohistochemistry (IHC)

Fixed tissues were embedded in paraffin, sectioned at 4 μm, and mounted on slides. After deparaffinization and antigen retrieval, tissues were blocked with 3% serum, followed by overnight incubation with a primary anti-BDNF antibody (1:200) [EPR1292] (ab108319) at 4°C. After rinsing, the HRP-labeled secondary antibody was incubated at 37°C for 20 min. Positive signals appeared as brownish-yellow deposits using DAB substrate, counterstained with hematoxylin, and imaged under a light microscope. The percentage of positive area was quantified using ImageJ software, with PBS as a negative control. Four to five fields were evaluated at ×200 and ×400 magnification.

#### Western blotting analysis

Tissues were prepared in RIPA buffer (50 mM Tris HCl, 1 mM EDTA, 150 mM NaCl, 1% Triton, 2% sodium dodecyl sulfate (SDS), and phosphatase and protease inhibitors), and clarified by centrifugation. Equal amounts of protein ran on SDS-polyacrylamide gels, transferred to polyvinylidene difluoride membranes, blocked in 3% bovine serum albumin, and incubated with the primary antibodies. Then appropriate secondary antibodies were used before development with an enhanced chemiluminescence reagent. The primary antibodies were as follows: BDNF [EPR1292] (ab108319) (1:1,000, Abcam), GAPDH (GB15004-100) (1:5,000, Servicebio).

#### Realtime PCR

RNA from cells or kidney tissues was isolated using TRIzol (Takara, Kyoto, Japan) and then reverse transcribed. The primer sequences were as follows: Gadph, primer F, 5′-CTGGGCTACACTGAGCACC-3′ and primer R, 5′-AAGTGGTCGTTGAGGGCAATG -3’. *Bdnf-as,* primer F, 5′-TTGACACACCAGAGAAGACACAC-3′ and primer R, 5′-GCCTGCTAAAGCCTCTACCA-3′. Real-time PCR was performed using SYBR Green PCR Master Mix (Vazyme, Nanjing, China) and the Rotor-Gene 3000A real-time PCR system (Corbett, Sydney, Australia) according to the manufacturer’s instructions. In brief, the PCR amplification reaction mixture (20 mL) contained 2 mL cDNA, 0.4 mL F primer, 0.4 mL R primer, and 10 mL SYBR Green I. After initial denaturation at 95°C for 1 min, the reaction was cycled 45 times. Each cycle consisted of denaturation at 95°C for 15 s and primer annealing and extension at 60°C for 31 s. Results are shown as the relative expression of *Bdnf-as* normalized to the expression of Gadph. Real-time PCR was performed in triplicate for each experiment, and the average values were measured. Each experiment was repeated three times. Using the gene-specific efficiencies, mRNA relative expression folds were calculated as 2^−ΔΔ^ circle threshold.

#### Single-cell sequencing

We performed single-cell RNA sequencing analysis using publicly available data from the Kidney Interactive Transcriptomics (KIT) database (https://www.humphreyslab.com/SingleCell/), with permission from Dr. Humphreys’ laboratory, to investigate transcriptomic differences between 14-day UUO mice and healthy controls. In parallel, expression analysis of *Bdnf-as* was conducted using human kidney transcriptomic data from the Kidney Precision Medicine Project (KPMP, https://atlas.kpmp.org/), focusing on differences between healthy individuals and CKD patients. Particular attention was given to cell type-specific gene expression patterns in both datasets.

#### Statistical analysis

The meta-analysis was conducted using Review Manager (RevMan) software version 5.4 (The Cochrane Collaboration), with data pooled using a random-effect model. Statistical heterogeneity between studies was quantitatively assessed through the *I* ^*2*^ statistic. When *I* ^*2*^ < 25%, there was low heterogeneity; when 25% < *I* ^*2*^ < 75%, there was moderate heterogeneity; and when *I* ^*2*^ > 75%, there was high heterogeneity (Higgins et al., 2003). Publication bias was evaluated through a combination of funnel plot asymmetry analysis and Egger’s linear regression test (Zwe et al., 2017). The funnel plot provided a visual assessment of potential bias by examining the distribution of effect sizes against their standard errors, while Egger’s test statistically quantified the likelihood of small-study effects (intercept significance: *P* < 0.10 indicating potential bias) (Lin et al., 2018). IHC data are presented as mean ± standard deviation (SD). Differences between groups were analyzed using one-way ANOVA in GraphPad Prism 8.0. A *P* < 0.05 was considered statistically significant.

## Results

### Meta-analysis

**FIGURE 1:**
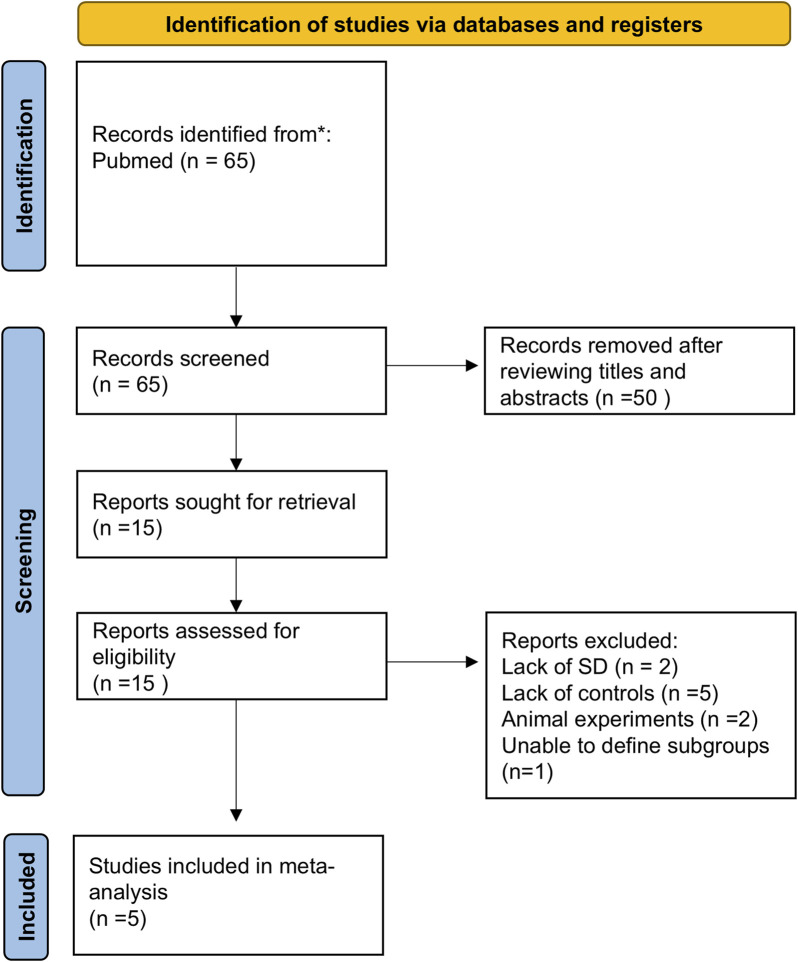
Flowchart of literature screening and exclusion process in meta-analysis. A total of 65 records were initially identified through systematic literature searches. After title and abstract screening, 50 records were excluded. The remaining 15 full-text articles underwent eligibility assessment, with 10 excluded due to: missing standard deviation data (n = 2), absence of control groups (n = 5), animal experiments (n = 2), and inability to define subgroups (n = 1). Three studies were ultimately included for quantitative synthesis. Abbreviations: SD, Standard Deviation.

An initial search identified 65 studies. After screening titles and abstracts, 50 were excluded, leaving 5 studies that met the inclusion criteria (Figure 1). The characteristics of these studies are detailed in Table 1. These 5 studies included two from European populations and three from Asian populations. Notably, while most studies were cross-sectional, the study by Kurajoh et al. (2017) was cohort-based. The sample source was primarily serum, with one study using plasma. Supplementary Table S3 presented the AHRQ assessment results for the selected studies.

**TABLE 1:** Characteristics of studies included in the meta-analysis.

First author	Year	Country	Type of study	Case/Control	Subjects (case/control)	Subjects (Male/Female)	Mean age±SD (yr) (case/control)	Mean BDNF±SD ng/mL (case/control)	CKD definition	Sample
Chiang	2024	Asian	cross-sectional study	CKD/non-CKD	156/392	312/236	69.70 ± 9.00/60.40 ± 8.50	21.40 ± 7.41/25.90 ± 6.96	CKD was defined as a condition with an eGFR <60 mL/min/1.73 m^2^	Serum
Gliwińska	2024	Europe	cross-sectional study	CKD/non-CKD	28/44	43/29	NA	0.64 ± 0.14/1.02 ± 0.23	Follow the 2012 KDIGO (Kidney Disease: Improving Global Outcomes) guidelines	Serum
Hsu, C Y	2023	Asian	cross-sectional study	CKD/non-CKD	87/393	393/87	69/58	21.10 ± 7.40/25.20 ± 8.80	Participants with CKD younger than 60 years of age into the case group and participants without CKD 60 years of age and into the control group	Serum
Lee	2018	Asian	cross-sectional study	CKD/non-CKD	60/65	54/71	50.53 ± 10.43/48.03 ± 9.35	11.06 ± 1.07/11.23 ± 1.18	1) between the ages of 20 and 64 years and 2) a diagnosis of CKD lasting more than 3 months	Serum
Kurajoh	2017	Europe	cross-sectional study	CKD/non-CKD	38/286	176/148	64.11 ± 9.00/57.04 ± 13.26	2.32 ± 1.95/2.96 ± 2.44	Development of CKD was defined as a decline in eGFR to less than 60 mL/min/1.73m^2^	Plasma

Abbreviations: CKD, chronic kidney disease; SD, standard deviation; eGFR, estimated glomerular filtration rate.

**FIGURE 2:**
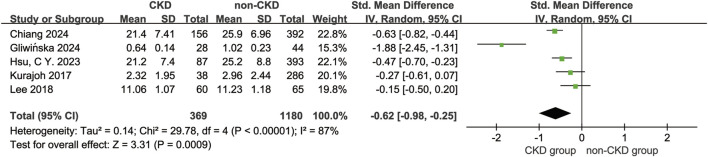
Forest plot of the Meta-Analysis. Forest plot summarizing SMDs from four studies (Chiang et al., 2024; Gliwińska et al., 2024; Hsu 2023; Lee et al., 2018; Kurajoh et al., 2017) using a random-effect inverse variance model. Diamond markers denote 95% confidence intervals; filled diamonds represent individual studies, and the open diamond indicates the overall pooled effect. Weight percentages reflect study contribution to the meta-analysis.

This meta-analysis compared serum BDNF levels between CKD patients (n = 369) and non-CKD controls (n = 1,180) using a random-effects model. The pooled effect size demonstrated a statistically significant reduction in serum BDNF levels among CKD patients (WMD = −0.62 ng/mL, 95% CI [−0.98, −0.25], *P* < 0.001), accompanied by substantial heterogeneity across studies (*I* ^*2*^ = 87%, *P* < 0.001) (Figure 2). Assessment of publication bias using funnel plot symmetry and Egger’s test (β = 0.682, 95% CI [–3.53, 4.90], *P* = 0.405) revealed no evidence of significant publication bias (Supplementary Figure S1). Sensitivity analysis, performed by sequentially removing each study, consistently confirmed the robustness of the main findings. Notably, heterogeneity was markedly reduced (*I* ^*2*^ = 60%) when the study by Gliwińska et al. (2024) was excluded, suggesting this study may contribute disproportionately to between-study variability, possibly due to its relatively small sample size.

### Progression of BDNF expression in the UUO model

**FIGURE 3:**
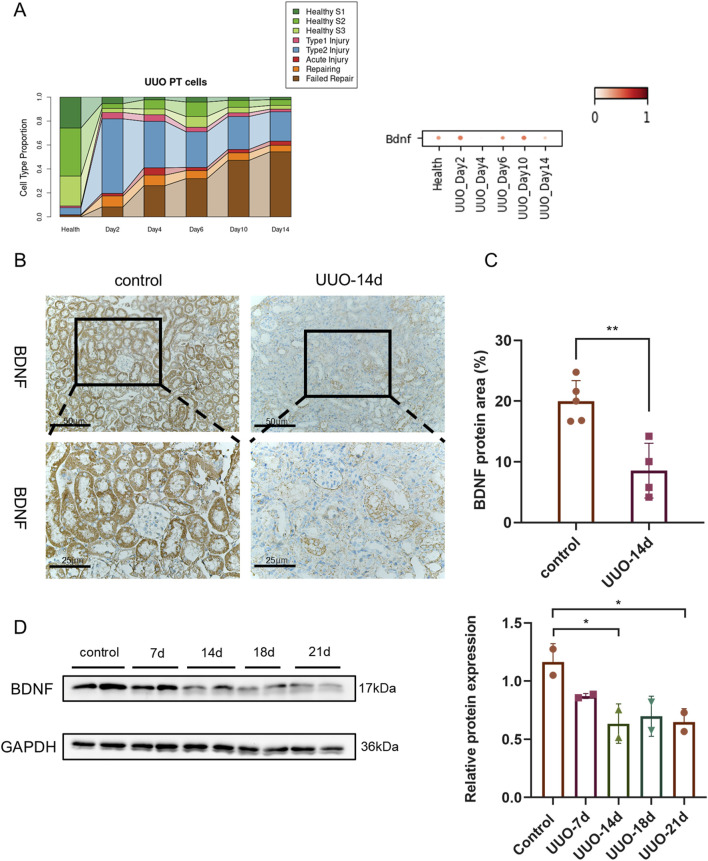
Progressive downregulation of BDNF protein in the obstructed kidney following UUO. **(A)** Analysis of cell type proportions and *Bdnf* gene expression in UUO-induced renal injury model. **(B)** Immunohistochemical staining of kidney tissues from control mice and UUO 14-day model mice at 200X and 400X microscopes showing brown staining results for BDNF protein. Where the image under ×400 microscope is a magnified view of the image under ×200 microscope. **(C)** Expression levels of BDNF proteins were further assessed by quantifying the percentage area of BDNF proteins in each group. **(D)** Western blot analysis of whole-kidney lysates harvested at 7, 14, 18 and 21 d post-UUO and control groups. GAPDH (36 kDa) served as internal Control. Densitometric quantification (normalized to GAPDH) is shown below the blot; n = 2 per time point; *P < 0.05, **P < 0.01.

As illustrated in Figure 3, single-cell transcriptomic analysis revealed a marked reduction in Bdnf mRNA expression in the kidneys of UUO-14day mice compared to normal controls (Figure 3A). This downregulation coincided with a shift in cellular composition, characterized by a decline in normal renal cells and a corresponding increase in damaged and unsuccessfully repaired cell populations. Immunohistochemical staining (Figures 3B,C) further confirmed the diminished BDNF protein expression in UUO-14 d kidneys. Western blot analysis (Figure 3D) revealed a progressive decline in BDNF protein levels in UUO kidneys at days 14, 18, and 21, with the steepest reduction observed at day 21.

### *Bdnf* downregulation and *Bdnf-as* upregulation in CKD

**FIGURE 4:**
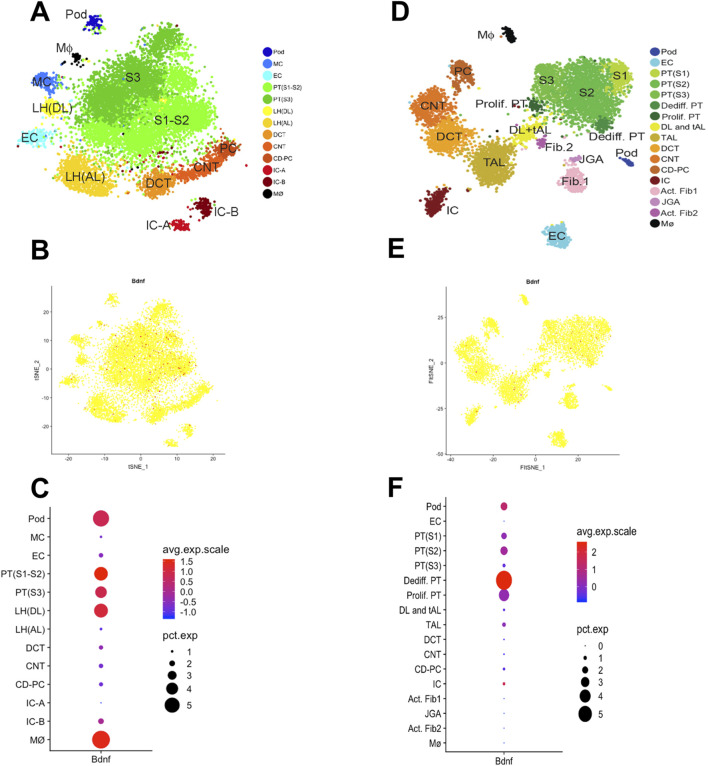
Single-cell transcriptomic analysis reveals kidney cell types and gene expression characteristics in mouse. **(A)** t-SNE downscaling plot showing the clustering distribution of healthy murine kidney cells. Different colors represent different cell types. **(B)** Distribution of *Bdnf* gene expression in healthy murine kidney cells, the color indicates the level of expression. **(C)** Average expression of *Bdnf* gene in different cell types (color indicated) and proportion of expressed cells (dot size). **(D)** t-SNE downscaling plot showing the clustering distribution of kidney cells in UUO 14-day mice. Different colors represent different cell types. **(E)** Distribution of *Bdnf* gene expression in UUO 14-day kidney cells. **(F)** Average expression (color representation) and proportion of expressed cells (dot size) of *Bdnf* gene in different cell types.

**FIGURE 5:**
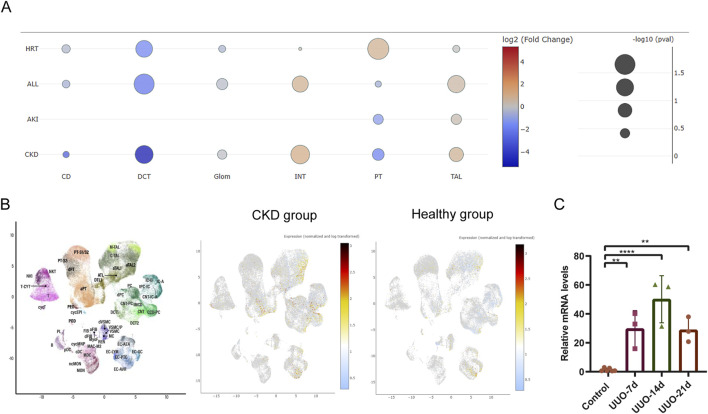
Single-cell transcriptomics and real-time PCR analysis of Bdnf-as cell type-specific expression dynamics in UUO kidneys and CKD humans. **(A)** Differential expression analysis of *bdnf* in various disease types under different disease states. Each bubble represents the expression of *bdnf* in specific cell types (CD, DCT, Glom, INT, PT, TAL) under different disease states (HRT, ALL, AKI, CKD). The color gradient indicates the log2 fold change (log2(FC)) in *bdnf* expression, with red representing upregulation and blue representing downregulation. Bubble size corresponds to the statistical significance of the change in expression. **(B)** scRNA-seq analysis of renal cells from healthy and CKD individuals. The UMAP plot shows the distribution of different cell clusters, with each cluster annotated by cell type markers. Expression of *Bdnf-as* gene in renal cells visualized by scRNA-seq. The color gradient represents normalized and log-transformed expression levels, with warmer colors (yellow/red) indicating higher expression. The plot demonstrates the expression pattern of *Bdnf-as* in healthy human and CKD group. **(C)** Real-time fluorescent quantitative PCR (qRT-PCR) validation. Total RNA was extracted from UUO mouse and control group kidneys, with Gapdh as the internal control, and the relative expression level of Bdnf-as was determined using the 2^^–ΔΔ^Ct method.

To further assess the cell-specific expression of the *Bdnf* gene and *Bdnf-as* gene, we analyzed single-cell RNA sequencing data from kidney tissues. The results showed that cell clustering in healthy mice was categorized into 12 major cell types (Figure 4A). In healthy mice (Figures 4B,C), *Bdnf* expression was mainly concentrated in the cell types of descending medullary collaterals (LH (DL)), proximal tubules (PT S1-S3), podocytes (Pod) and macrophages (Mφ). Whereas cell clustering in UUO14-day mice was further subdivided into 14 cell types (Figure 4D). *Bdnf* levels were significantly upregulated in dedifferentiated PT cells in UUO14-day mice and were significantly downregulated in PT (S1-3) cells compared to healthy groups (Figures 4E,F). In the analysis of population transcriptome databases, we observed a trend of elevated *Bdnf* expression in renal interstitium (Figure 5A). Based on this finding, we further investigated in depth the changes in the levels of *Bdnf-as* which may play a role in regulating *Bdnf* expression. The results showed that the expression of *Bdnf-as* was also elevated in the proximal tubules of CKD patients (Figure 5B). As shown in Figure 5C, real-time qPCR in UUO mice confirmed the trend observed in human single-cell RNA-seq data—namely, a significant and progressive increase in Bdnf-as expression in kidney tissue at days 14 and 18 post-obstruction compared to controls (*P* < 0.01).

## Discussion

In this study, we employed a comprehensive, multi-tiered approach—encompassing meta-analysis of clinical data, experimental validation using a murine UUO model, and single-cell transcriptomic profiling—to investigate the relationship between CKD and *BDNF* expression. Our findings consistently demonstrated a significant downregulation of BDNF at serum, renal protein and transcriptomic levels in CKD settings. Specifically, immunohistochemistry revealed markedly decreased *BDNF* expression in the kidneys of UUO mice, supporting a link between renal injury and *BDNF* depletion. Furthermore, single-cell RNA sequencing identified substantial reductions in *BDNF* transcripts in key renal cell populations, such as proximal tubule cells, podocytes, and macrophages, in UUO models compared to healthy controls. Transcriptomic analysis also showed that *Bdnf-as* gene expression was significantly unregulated in CKD proximal tubule cells. Complementing these experimental observations, our meta-analysis of human studies confirmed a robust and statistically significant decrease in circulating BDNF levels among CKD patients.

BDNF is a neurotrophic factor with broad biological functions, existing in two distinct molecular forms: the precursor form (pro-BDNF) and the mature form (m-BDNF) (Angelucci et al., 2019). These isoforms exert opposing effects via different receptor pathways. Pro-BDNF primarily binds to the p75 neurotrophin receptor (p75^NTR), triggering pro-apoptotic signaling cascades that can lead to cell death (Azman and Zakaria, 2022). In contrast, m-BDNF engages with the high-affinity TrkB receptor, mediating classical neurotrophic functions including the promotion of neuronal survival, synaptic plasticity, and cellular resilience. Beyond its well-established role in the nervous system, m-BDNF is increasingly recognized as a pleiotropic molecule involved in various peripheral physiological processes, such as myocardial ischemia protection (Cannavo et al., 2023), promotion of angiogenesis (Mori et al., 2018), and regulation of skeletal muscle metabolism (Yang et al., 2019). Intriguingly, enhanced *BDNF* mRNA expression and upregulated TrkB receptor levels have been reported in podocytes of patients with diabetic nephropathy (Endlich et al., 2018), suggesting a possible compensatory or stress-response mechanism in hyperglycemic renal environments. However, our single-cell transcriptomic data revealed a contrasting pattern: podocyte-specific downregulation of *BDNF* expression in UUO mice. This discrepancy may reflect fundamental mechanistic differences between hyperglycemia-induced damage and obstructive injury. In diabetic nephropathy, persistent high-glucose conditions may induce BDNF upregulation as an adaptive response, whereas the UUO model is more likely associated with fibrotic progression, oxidative stress, and mechanical injury that suppresses *BDNF* transcription.

Further supporting this hypothesis, prior studies have shown divergent trends in circulating BDNF levels in metabolic diseases. While some investigations reported reduced serum BDNF in patients with type 2 diabetes mellitus (He et al., 2024), Sparta et al. (2019) observed an initial elevation during early disease stages, followed by a decline as the disease progressed—this may be related to impaired BDNF compensatory mechanisms caused by chronic oxidative stress and systemic inflammation. In line with this, BDNF suppression was also observed in animal models after administration of uremic toxins such as indoxyl sulfate (Sun et al., 2021) and p-cresyl sulfate (Sun et al., 2020), indicating that CKD-related metabolic disruptions may impair BDNF synthesis or release.

Notably, clinical improvement of renal function has been linked to BDNF restoration. For instance, patients with end-stage renal disease demonstrated a significant increase in serum BDNF levels 2 years after renal transplantation (Hernandez et al., 2023), indirectly supporting a positive correlation between renal function and systemic BDNF status. Conversely, Zoladz et al. (2012) reported a transient reduction in serum BDNF following hemodialysis, likely attributable to acute stress and inflammatory activation rather than long-term trends.

At the molecular level, several intracellular signaling pathways are known to regulate *BDNF* transcription, including lncRNA *Bdnf-as* (Modarresi et al., 2012), calcium signaling (Fukuchi et al., 2005; Qiu et al., 2020), Wnt/β-catenin cascade (Lou et al., 2024), and CREB phosphorylation (Amidfar et al., 2020). Recent evidence also suggests that inhibition of the AhR/NF-κB/JNK axis activates BDNF/TrkB signaling, which attenuates the progression of CKD and the cognitive deficits associated with CKD (Gu et al., 2022). However, due to BDNF’s short half-life and difficulty in crossing the blood-brain barrier, it is not possible to directly inject BDNF molecules to increase their levels. Currently, the main agonists used to enhance BDNF signaling include TrkB agonists and BDNF mimetic molecules (Alqahtani et al., 2025). These agonists were valuable in the treatment of Alzheimer’s disease. In addition, some drugs commonly used in clinical practice, such as statins and metformin, have also been found to work by activating the BDNF signaling pathway (Xu et al., 2022). Research has found that diabetic patients receiving metformin treatment have significantly elevated serum BDNF levels (He et al., 2024). Although the currently available BDNF agonists have achieved a certain degree of efficacy, they still suffer from low bioavailability and poor pharmacological properties, and need to be further optimized and improved. Kamel et al. (2025) found that the use of DDP-4 inhibitors can enhance BDNF/TrkB/NRF2 signaling, thereby improving acute kidney injury. Mechanistically, *Bdnf-as* is a long-chain non-coding RNA that has been found to inhibit BDNF expression by altering *BDNF* chromatin conformation and methylation (Modarresi et al., 2012). Silencing of *Bdnf-as* has been found to reduce apoptosis by regulating BDNF/TrkB/PI3K/Akt signaling (Xu et al., 2022) as well as affect AD progression. Our transcriptomics study showed that the *Bdnf-as* gene was significantly upregulated in the proximal renal tubules of CKD, whereas BDNF expression was decreased in CKD, suggesting that it may play an important role in the regulation of BDNF expression in CKD. These mechanistic studies provide a good direction for the therapeutic regulation of BDNF in CKD and its related complications.

Our study advances prior work by uncovering the tissue-level and regulatory mechanisms behind BDNF loss in CKD. While earlier studies reported reduced circulating BDNF, they lacked insight into local renal expression or upstream drivers. This is the first study to integrate meta-analysis, single-cell transcriptomics, and epigenetic profiling to reveal cell-specific BDNF downregulation and identify *Bdnf-as* as a novel CKD-associated lncRNA that may epigenetically suppress BDNF. Although this study combined multiple methods for validation, shortcomings remain. First, the study by Gliwińska et al. demonstrated a high degree of heterogeneity (*I* ^*2*^ = 87%), which may be attributed to the small sample size of the study and the inclusion of a population of Polish children with a large age span (1.17–18 years). This feature differs from several other studies that included data from adults, where subjects tended to have comorbidities such as diabetes mellitus and hypertension, whereas the etiology of CKD in children is usually associated with congenital malformations. Secondly, although a negative correlation between BDNF levels and CKD severity was observed, the causal relationship between BDNF depletion and CKD progression needs to be functionally examined by gain-of-function/loss-of-function modeling in the future. Thirdly, the pathway mechanisms of *Bdnf-as* for epigenetic regulation have not been investigated in detail, and although single-cell histology suggests that *Bdnf-as* plays an important role, visual evidence is still needed to prove it. Nevertheless, the results of this study provide important implications for the translation of BDNF from the laboratory to the clinic. Fourth, while we observed a consistent decline in renal BDNF expression corresponding with disease progression in UUO mice, the current study design does not allow for a causal inference. Future studies utilizing functional models—such as conditional BDNF overexpression or knockout in kidney tissue—are needed to clarify whether BDNF loss actively contributes to CKD pathogenesis or is merely a consequence of injury.

## Conclusion

This study demonstrates a significant reduction of BDNF in CKD, both systemically and within the kidney. Renal BDNF expression was decreased in proximal tubule cells, macrophages, and podocytes, alongside upregulation of the inhibitory lncRNA *Bdnf-as*. These findings highlight BDNF downregulation as a potential contributor to CKD progression and a target for future intervention.

## Data Availability

The original contributions presented in the study are included in the article/Supplementary Material, further inquiries can be directed to the corresponding authors.
