# Supplementary figures and images for: Decreased brain-derived neurotrophic factor expression in chronic kidney disease: integrated clinical and experimental evidence
Source: Front Mol Biosci. 2025 Jul 28;12:1627534. doi: 10.3389/fmolb.2025.1627534 (PMC12336020; doi:10.3389/fmolb.2025.1627534)

## Slide 1
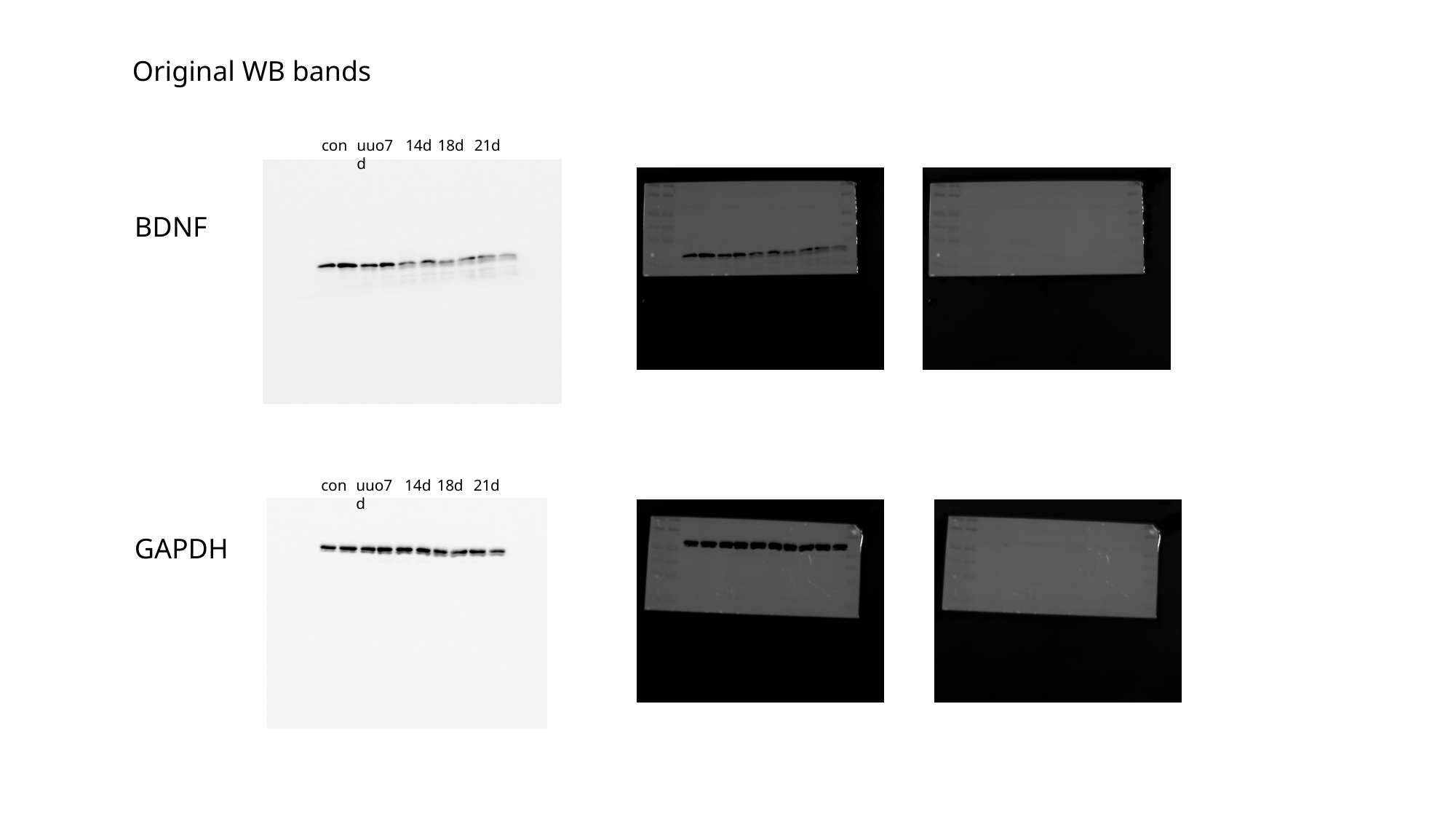

Original WB bands
18d
21d
uuo7d
14d
con
BDNF
18d
21d
uuo7d
14d
con
GAPDH

Supplement: Supplementary file 1 [file Presentation1.pptx]
