# Supplementary material for: Decreased brain-derived neurotrophic factor expression in chronic kidney disease: integrated clinical and experimental evidence
Source: Front Mol Biosci. 2025 Jul 28;12:1627534. doi: 10.3389/fmolb.2025.1627534 (PMC12336020; doi:10.3389/fmolb.2025.1627534)
Supplement: Supplementary file 2 [file DataSheet1.docx]

**Decreased Brain-Derived Neurotrophic Factor Expression in Chronic Kidney Disease: Integrated Clinical and Experimental Evidence**

Juan Chen^1(†)^, Lili Fu^1(†)^, Mengjin Li^1(†)^, Bo Yang^1^, Kun Xie^2^, Xinming Li^1^, Xu-jie Zhou^3^, Li Yang^3^, Nanmei Liu^1^, Liming Zhang^4^, Cheng Xue ^1 5^*, Zhiguo Mao^1^*

1 Department of Nephrology, Shanghai Changzheng Hospital, Second Affiliated Hospital of Naval Medical University, Second Military Medical University, Shanghai, China.

2 Department of Nephrology, Northern Jiangsu People’s Hospital Affiliated to Yangzhou University, Jiangsu, China.

3 Renal Division, Ministry of Health of China, Institute of Nephrology, Key Laboratory of Renal Disease, Peking University First Hospital, Peking University, Beijing, China.

4 Department of Nephrology, Zhabei Central Hospital of Jing'an District, Shanghai, China.

5 Department of Cellular Biology and Anatomy, Medical College of Georgia at Augusta University, Augusta, Georgia, United States.

^(^†^)^ These authors contributed equally to this work.

**Correspondence** Zhiguo Mao, or Cheng Xue, Division of Nephrology, Shanghai Changzheng Hospital, Second Military Medical University (Naval Medical University), 415 Fengyang Road, Shanghai, 200003, China. Tel: +86-21-81885005, Fax: +86-21-81885005, Email: [maozhiguo518@126.com](mailto:maozhiguo518@126.com), or chengxia1568@126.com/cxue@augusta.edu.

**Supplementary Data**

**Supplementary Table 1. Literature Search Strategy in PubMed**

**Supplementary table 2. Preferred Reporting Items for Systematic Reviews and Meta-Analyses statement.**

**Supplementary table 3. Quality Assessment of cross-sectional study Studies Using Agency for Healthcare Research and Quality Scores**

**Supplementary Figure 1. Funnel Plots of the Meta-Analysis.**

**Supplementary Table 1. Literature Search Strategy in PubMed**

| PubMed |
| --- |
| *Chronic kidney disease* |
| 1 (((((((((((((((((((Renal Insufficiency, Chronic[MeSH Terms]) OR (Chronic Renal Insufficiencies)) OR (Renal Insufficiencies, Chronic)) OR (Chronic Kidney Insufficiency)) OR (Chronic Kidney Insufficiencies)) OR (Kidney Insufficiencies, Chronic)) OR (Chronic Renal Insufficiency)) OR (Kidney Insufficiency, Chronic)) OR (Chronic Kidney Diseases)) OR (Chronic Kidney Disease)) OR (Disease, Chronic Kidney)) OR (Diseases, Chronic Kidney)) OR (Kidney Disease, Chronic)) OR (Kidney Diseases, Chronic)) OR (Chronic Renal Diseases)) OR (Chronic Renal Disease)) OR (Disease, Chronic Renal)) OR (Diseases, Chronic Renal)) OR (Renal Disease, Chronic)) OR (Renal Diseases, Chronic) |
| (((((((((((((((((((Renal Insufficiency, Chronic[MeSH Terms]) OR (Chronic Renal Insufficiencies)) OR (Renal Insufficiencies, Chronic)) OR (Chronic Kidney Insufficiency)) OR (Chronic Kidney Insufficiencies)) OR (Kidney Insufficiencies, Chronic)) OR (Chronic Renal Insufficiency)) OR (Kidney Insufficiency, Chronic)) OR (Chronic Kidney Diseases)) OR (Chronic Kidney Disease)) OR (Disease, Chronic Kidney)) OR (Diseases, Chronic Kidney)) OR (Kidney Disease, Chronic)) OR (Kidney Diseases, Chronic)) OR (Chronic Renal Diseases)) OR (Chronic Renal Disease)) OR (Disease, Chronic Renal)) OR (Diseases, Chronic Renal)) OR (Renal Disease, Chronic)) OR (Renal Diseases, Chronic) |
| (((((((((((((((((((Renal Insufficiency, Chronic[MeSH Terms]) OR (Chronic Renal Insufficiencies)) OR (Renal Insufficiencies, Chronic)) OR (Chronic Kidney Insufficiency)) OR (Chronic Kidney Insufficiencies)) OR (Kidney Insufficiencies, Chronic)) OR (Chronic Renal Insufficiency)) OR (Kidney Insufficiency, Chronic)) OR (Chronic Kidney Diseases)) OR (Chronic Kidney Disease)) OR (Disease, Chronic Kidney)) OR (Diseases, Chronic Kidney)) OR (Kidney Disease, Chronic)) OR (Kidney Diseases, Chronic)) OR (Chronic Renal Diseases)) OR (Chronic Renal Disease)) OR (Disease, Chronic Renal)) OR (Diseases, Chronic Renal)) OR (Renal Disease, Chronic)) OR (Renal Diseases, Chronic) |
| (((((((((((((((((((Renal Insufficiency, Chronic[MeSH Terms]) OR (Chronic Renal Insufficiencies)) OR (Renal Insufficiencies, Chronic)) OR (Chronic Kidney Insufficiency)) OR (Chronic Kidney Insufficiencies)) OR (Kidney Insufficiencies, Chronic)) OR (Chronic Renal Insufficiency)) OR (Kidney Insufficiency, Chronic)) OR (Chronic Kidney Diseases)) OR (Chronic Kidney Disease)) OR (Disease, Chronic Kidney)) OR (Diseases, Chronic Kidney)) OR (Kidney Disease, Chronic)) OR (Kidney Diseases, Chronic)) OR (Chronic Renal Diseases)) OR (Chronic Renal Disease)) OR (Disease, Chronic Renal)) OR (Diseases, Chronic Renal)) OR (Renal Disease, Chronic)) OR (Renal Diseases, Chronic) |
| (((((((((((((((((((Renal Insufficiency, Chronic[MeSH Terms]) OR (Chronic Renal Insufficiencies)) OR (Renal Insufficiencies, Chronic)) OR (Chronic Kidney Insufficiency)) OR (Chronic Kidney Insufficiencies)) OR (Kidney Insufficiencies, Chronic)) OR (Chronic Renal Insufficiency)) OR (Kidney Insufficiency, Chronic)) OR (Chronic Kidney Diseases)) OR (Chronic Kidney Disease)) OR (Disease, Chronic Kidney)) OR (Diseases, Chronic Kidney)) OR (Kidney Disease, Chronic)) OR (Kidney Diseases, Chronic)) OR (Chronic Renal Diseases)) OR (Chronic Renal Disease)) OR (Disease, Chronic Renal)) OR (Diseases, Chronic Renal)) OR (Renal Disease, Chronic)) OR (Renal Diseases, Chronic) |
| (((((((((((((((((((Renal Insufficiency, Chronic[MeSH Terms]) OR (Chronic Renal Insufficiencies)) OR (Renal Insufficiencies, Chronic)) OR (Chronic Kidney Insufficiency)) OR (Chronic Kidney Insufficiencies)) OR (Kidney Insufficiencies, Chronic)) OR (Chronic Renal Insufficiency)) OR (Kidney Insufficiency, Chronic)) OR (Chronic Kidney Diseases)) OR (Chronic Kidney Disease)) OR (Disease, Chronic Kidney)) OR (Diseases, Chronic Kidney)) OR (Kidney Disease, Chronic)) OR (Kidney Diseases, Chronic)) OR (Chronic Renal Diseases)) OR (Chronic Renal Disease)) OR (Disease, Chronic Renal)) OR (Diseases, Chronic Renal)) OR (Renal Disease, Chronic)) OR (Renal Diseases, Chronic) |
| **#1**: 1 |
| *BDNF*  2 ((((Brain-Derived Neurotrophic Factor [MeSH Terms]) OR (Brain Derived Neurotrophic Factor)) OR (Factor, Brain-Derived Neurotrophic)) OR (Neurotrophic Factor, Brain-Derived)) OR (BDNF)  **#2***: 2* |
| Combined search: #1 and #2 (language limit to English) |

**Supplementary table 2. Preferred Reporting Items for Systematic Reviews and Meta-Analyses statement.**

| **Section and Topic** | **Item** | **Checklist item** | **Location where item is reported** |
| --- | --- | --- | --- |
| **TITLE** | | |  |
| Title | 1 | Identify the report as a systematic review. | 1 |
| **ABSTRACT** | | |  |
| Abstract | 2 | See the PRISMA 2020 for Abstracts checklist. | 3, supplementary table 2 |
| **INTRODUCTION** | | |  |
| Rationale | 3 | Describe the rationale for the review in the context of existing knowledge. | 3-4 |
| Objectives | 4 | Provide an explicit statement of the objective(s) or question(s) the review addresses. | 3-4 |
| **METHODS** | | |  |
| Eligibility criteria | 5 | Specify the inclusion and exclusion criteria for the review and how studies were grouped for the syntheses. | 3-4 |
| Information sources | 6 | Specify all databases, registers, websites, organisations, reference lists and other sources searched or consulted to identify studies. Specify the date when each source was last searched or consulted. | Figure 1 |
| Search strategy | 7 | Present the full search strategies for all databases, registers and websites, including any filters and limits used. | 3-5, supplementary table 3 |
| Selection process | 8 | Specify the methods used to decide whether a study met the inclusion criteria of the review, including how many reviewers screened each record and each report retrieved, whether they worked independently, and if applicable, details of automation tools used in the process. | 3-4 |
| Data collection process | 9 | Specify the methods used to collect data from reports, including how many reviewers collected data from each report, whether they worked independently, any processes for obtaining or confirming data from study investigators, and if applicable, details of automation tools used in the process. | 3-4 |
| Data items | 10a | List and define all outcomes for which data were sought. Specify whether all results that were compatible with each outcome domain in each study were sought (e.g. for all measures, time points, analyses), and if not, the methods used to decide which results to collect. | 3-4, 7, Table 1 |
|  | 10b | List and define all other variables for which data were sought (e.g. participant and intervention characteristics, funding sources). Describe any assumptions made about any missing or unclear information. | 7, Table 1 |
| Study risk of bias assessment | 11 | Specify the methods used to assess risk of bias in the included studies, including details of the tool(s) used, how many reviewers assessed each study and whether they worked independently, and if applicable, details of automation tools used in the process. | 3-4, 6, supplementary table 3 |
| Effect measures | 12 | Specify for each outcome the effect measure(s) (e.g. risk ratio, mean difference) used in the synthesis or presentation of results. | 7, Figure 2 |
| Synthesis methods | 13a | Describe the processes used to decide which studies were eligible for each synthesis (e.g. tabulating the study intervention characteristics and comparing against the planned groups for each synthesis (item #5)). | 4, 7,  supplementary table 3 |
|  | 13b | Describe any methods required to prepare the data for presentation or synthesis, such as handling of missing summary statistics, or data conversions. | 4, 7 |
|  | 13c | Describe any methods used to tabulate or visually display results of individual studies and syntheses. | 7, Table 1, Figure 2 |
|  | 13d | Describe any methods used to synthesize results and provide a rationale for the choice(s). If meta-analysis was performed, describe the model(s), method(s) to identify the presence and extent of statistical heterogeneity, and software package(s) used. | 6-7 |
|  | 13e | Describe any methods used to explore possible causes of heterogeneity among study results (e.g. subgroup analysis, meta-regression). | 6-7, supplementary figure 1 |
|  | 13f | Describe any sensitivity analyses conducted to assess robustness of the synthesized results. | 6-7, supplementary figure 1 |
| Reporting bias assessment | 14 | Describe any methods used to assess risk of bias due to missing results in a synthesis (arising from reporting biases). | 6-7, supplementary figure 1 |
| Certainty assessment | 15 | Describe any methods used to assess certainty (or confidence) in the body of evidence for an outcome. | 6-7 |
| **RESULTS** | | |  |
| Study selection | 16a | Describe the results of the search and selection process, from the number of records identified in the search to the number of studies included in the review, ideally using a flow diagram. | 4,  Figure 1 |
|  | 16b | Cite studies that might appear to meet the inclusion criteria, but which were excluded, and explain why they were excluded. | 8-10 |
| Study characteristics | 17 | Cite each included study and present its characteristics. | 7, Table 1 |
| Risk of bias in studies | 18 | Present assessments of risk of bias for each included study. | 7 |
| Results of individual studies | 19 | For all outcomes, present, for each study: (a) summary statistics for each group (where appropriate) and (b) an effect estimates and its precision (e.g. confidence/credible interval), ideally using structured tables or plots. | 7, Figure 2 |
| Results of syntheses | 20a | For each synthesis, briefly summarise the characteristics and risk of bias among contributing studies. | 7 |
|  | 20b | Present results of all statistical syntheses conducted. If meta-analysis was done, present for each the summary estimate and its precision (e.g. confidence/credible interval) and measures of statistical heterogeneity. If comparing groups, describe the direction of the effect. | 7 |
|  | 20c | Present results of all investigations of possible causes of heterogeneity among study results. | 7 |
|  | 20d | Present results of all sensitivity analyses conducted to assess the robustness of the synthesized results. | 7, supplementary figure 1 |
| Reporting biases | 21 | Present assessments of risk of bias due to missing results (arising from reporting biases) for each synthesis assessed. | 7, supplementary figure 1 |
| Certainty of evidence | 22 | Present assessments of certainty (or confidence) in the body of evidence for each outcome assessed. | Supplementary table 3 |
| **DISCUSSION** | | |  |
| Discussion | 23a | Provide a general interpretation of the results in the context of other evidence. | 9-10 |
|  | 23b | Discuss any limitations of the evidence included in the review. | 10-11 |
|  | 23c | Discuss any limitations of the review processes used. | - |
|  | 23d | Discuss implications of the results for practice, policy, and future research. | 10-11 |
| **OTHER INFORMATION** | | |  |
| Registration and protocol | 24a | Provide registration information for the review, including register name and registration number, or state that the review was not registered. | - |
|  | 24b | Indicate where the review protocol can be accessed, or state that a protocol was not prepared. | - |
|  | 24c | Describe and explain any amendments to information provided at registration or in the protocol. | - |
| Support | 25 | Describe sources of financial or non-financial support for the review, and the role of the funders or sponsors in the review. | **funding** |
| Competing interests | 26 | Declare any competing interests of review authors. | Competing interest |
| Availability of data, code and other materials | 27 | Report which of the following are publicly available and where they can be found: template data collection forms; data extracted from included studies; data used for all analyses; analytic code; any other materials used in the review. | Data availability |

**Supplementary table 3**. **Quality Assessment of cross-sectional study Studies Using Agency for Healthcare Research and Quality Scores**

| Study | Scoring Items | | | | | | | | | | | Scores | Quality ^b^ |
| --- | --- | --- | --- | --- | --- | --- | --- | --- | --- | --- | --- | --- | --- |
|  | 1 ^a^ | 2 ^a^ | 3 ^a^ | 4 ^a^ | 5 ^a^ | 6 ^a^ | 7 ^a^ | 8 ^a^ | 9 ^a^ | 10 ^a^ | 11 ^a^ |  |  |
| Chiang, 2024 | Yes | Yes | Yes | Yes | No | Yes | Yes | Yes | Unclear | Yes | Unclear | 9 | High |
| Gliwińska, 2024 | Yes | Yes | Yes | No | Yes | No | No | Yes | Yes | Yes | No | 6 | Median |
| Hsu, C Y., 2024 | Yes | No | Yes | Yes | No | Yes | Unclear | No | No | Yes | Unclear | 6 | Median |
| Lee, 2018 | Yes | Yes | Yes | No | No | Unclear | Unclear | No | No | Yes | Unclear | 4 | low |
| Kurajoh, 2017 | Yes | Yes | Yes | No | Yes | Yes | Yes | Yes | No | Yes | Yes | 8 | High |

a, 1, Define the source of information (survey, record review). 2, List inclusion and exclusion criteria for exposed and unexposed subjects (cases andcontrols) or refer to previous publications. 3, Indicate time period used for identifying patients. 4, Indicate whether or not subjects were consecutive if not population-based. 5, Indicate if evaluators of subjective components of study were masked to other aspects of the status of the participants. 6, Describe any assessments undertaken for quality assurance purposes (e.g., test/retest of primary outcome measurements). 7, Explain any patient exclusions from analysis. 8, Describe how confounding was assessed and/or controlled.  9, If applicable, explain how missing data were handled in the analysis. 10, Summarize patient response rates and completeness of data collection. 11, Clarify what follow-up, if any, was expected and the percentage of patients for which incomplete data or follow-up was obtained. b, Total stars awarded.

**Supplementary Figure 1. Funnel Plots of the Meta-Analysis.**


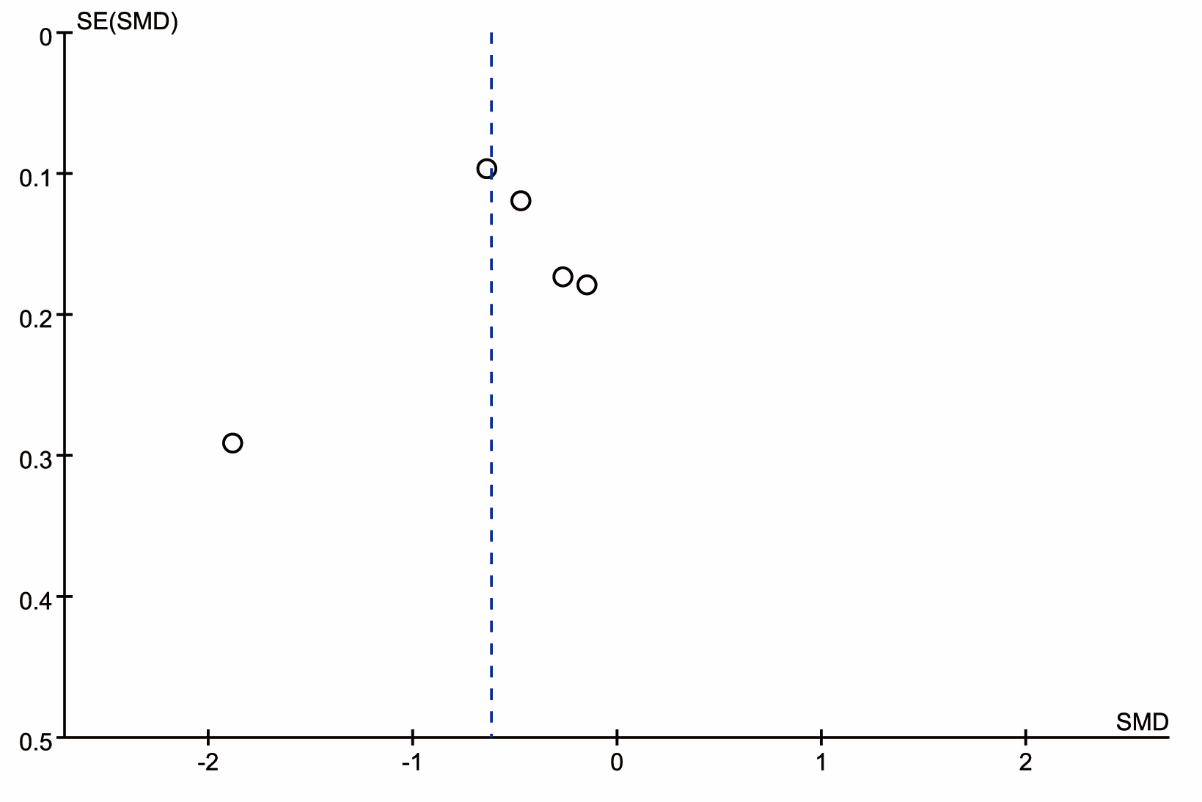


The horizontal axis (SMD) represents the Standardized Mean Difference (SMD) and the vertical axis (SE(SMD)) represents the standard error of the SMD. Egger test showed no evidence of publication bias (*P* = 0.405).
